# Lord Sumption and the values of life, liberty and security: before and since the COVID-19 outbreak

**DOI:** 10.1136/medethics-2021-107332

**Published:** 2021-07-12

**Authors:** John Coggon

**Affiliations:** Centre for Health, Law, and Society, University of Bristol Law School, Bristol, BS8 1HH, UK

**Keywords:** bills, laws and cases, coercion, COVID-19, end-of-life, philosophical ethics

## Abstract

Lord Sumption, a former Justice of the Supreme Court, has been a prominent critic of coronavirus restrictions regulations in the UK. Since the start of the pandemic, he has consistently questioned both the policy aims and the regulatory methods of the Westminster government. He has also challenged rationales that hold that all lives are of equal value. In this paper, I explore and question Lord Sumption’s views on morality, politics and law, querying the coherence of his broad philosophy and his arguments regarding coronavirus regulations with his judicial decision in the assisted-dying case of *R (Nicklinson) v Ministry of Justice*. In *Nicklinson*, Lord Sumption argued for restrictions on liberty given the priority of the sanctity of life principle and the protection of others who may be vulnerable, as well as for deference to policy-making institutions in instances of values-based disagreement. The apparent inconsistencies in his positions, I argue, are not clearly reconcilable, and invite critical analysis of his impacts on health law and policy.

## Introduction

Public health is a value-laden field. As a vocation and practice, it rests on moral mandates.^1 2^ As a matter of politics and government, it hangs on however we might answer the philosophical question ‘what makes health public?’; our explanations of whether, why and in what ways health and health-affecting phenomena are a shared concern within a community.^3^ And as a matter of law and regulation, the legitimacy of public health policy rests on the relationships—and tensions—between legal mandates that support governance for the public’s health, legal principles that constrain government activity (eg, the rule of law, principles of equality and respect for human rights), and the conformity between these values and wider principles of social justice.^4–7^

Because questions of ethical value pervade public health practice and policy, they necessarily predominate government responses to the coronavirus pandemic; for better or worse, and to whatever extent this may be explicitly acknowledged. Such questions demand ethical analysis and justification both of the proper aims of policy, and of the proper means of achieving those aims.^8 9^ Nevertheless, in the UK, there has been significant criticism of the paucity of ethical discourse at the level of government; early into the initial responses,^10^ and more recently.^11^ The breadth of the ethical challenges, the costs and the ethical trade-offs have tremendous and complex reach.^12^ It is essential to have open, public debate on the values that will and will not determine policy.

In public spheres beyond government, values have been raised and debated. Perhaps inevitably, given the nature and prominence of different platforms for public discourse, particular voices and views have found particularly privileged place in critical discussions of pandemic responses in the UK. Among these are the arguments of Lord Sumption, a former Justice of the Supreme Court. Lord Sumption is an eloquent and vocal critic of the restrictions regulations (and related points of official guidance) that have been implemented since March 2020. These regulations have significantly curtailed freedoms, including through the institution of protracted lockdowns, to limit transmission of the virus and, in turn, to prevent the National Health Service (NHS) from being overwhelmed.^13^ Lord Sumption has consistently questioned the moral, political and legal legitimacy of the measures, challenging both their underpinning rationales—their aims—and the justifiability of their form—the means of achieving those aims.

In what follows, I critique and question Lord Sumption’s ethical arguments about legal responses to the coronavirus pandemic. I do so against other aspects of Lord Sumption’s own reasoning, as advanced both in his role as a social commentator speaking about law and political morality, and as a senior judge; a position that allowed him firmly to influence the place and impact of moral values within the body of law. In the next section, I explain how Lord Sumption has been a forceful advocate, as a social commentator, for libertarian ideas and ideals. He argues that the overall reach of government responsibility is too great, and wrongly poised towards questions of safety and security over liberty. He also argues that within the reach of public decisions, too many questions have been made into ones that may be addressed by and through recourse to the law. However, as the subsequent part of the paper explains, as a judge in the UK Supreme Court, in the case of *R (Nicklinson) v Ministry of Justice*,^14^ Lord Sumption needlessly advanced moral arguments that prioritise the principle of the sanctity of life over autonomy, and which subjugate a defence of liberty to an imperative to protect people who might be considered vulnerable. I query the consistency between these positions and his broader philosophy. And in the final substantive section of the paper, I draw out the critique by considering Lord Sumption’s criticisms of coronavirus regulations against the backdrop—substantive and rhetorical—of his reasoning in *Nicklinson*. The dissonance between his reasoning both on the balancing of safety and liberty, and on the value of life, is not obviously reconcilable. I suggest that this seems to call into question his judgment in *Nicklinson*; something that would be particularly remarkable given his distaste for law’s encroachment, as he sees it, into the realms of politics and personal morality. Alternatively, it calls into question the quality of his reasoning about restrictions regulations. Either way, it suggests need for circumspection about Lord Sumption’s arguments as we evaluate the ethical challenges, balances and trade-offs that we face as we respond to the crisis.

## Lord Sumption on law, politics and morality

Lord Sumption is an extraordinary figure in UK law and public life. Even his route to becoming a Justice of the Supreme Court—being elevated directly to the UK’s highest court from his practice as a barrister—was far from typical.^15^ Both as a former senior judge and as a prominent commentator on law, government and social ethics, he is notable for his consistent advocacy for reconsidering the proper limits of law. His arguments may be seen to rest both on principled and practical grounds. His analysis speaks to boundaries on what law can and should do, and how far into spheres of decision-making it should reach; notably in relation to human rights jurisprudence and judicial review of decisions made by public bodies, and in regard to questions of individual morality.^16^

Consistent with this outlook, Lord Sumption is critical of the existing range of questions that may be decided through legal adjudication.^16^ Strikingly, however, we know this because he is at once both concerned about judicial overreach into what he argues to be the proper domain of politics, and one of the UK’s most politically outspoken judicial figures. This includes—as has been prominently seen throughout the coronavirus pandemic (on which more below)—being outspoken about what he sees as legislative overreach and excesses by government; he is a staunch critic of restrictions regulations. To explore that further, and critically against questionable inconsistencies with his position in the Nicklinson case, it is useful first to provide an overview of his general outlook on law, politics and morality.

For Lord Sumption, as he explained in his 2019 BBC Reith lectures^17^ and the subsequently published book *Trials of the State*,^18^ questions of personal morality on which there is no moral consensus have problematically become the property ‘of the community as a whole, as matters for collective and not personal decision’ (p 9).^18^ He exemplifies the point with reference to the Charlie Gard case (pp 9–12);^18 19^ a legal case concerning a tragic disagreement between doctors and parents on the best interests of a severely ill baby, which has been the subject of significant debate in UK bioethics.^20 21^ Lord Sumption laments the expansion of what came to fall within the reach of public decision-making during the 20th century, especially since the founding of the welfare state after the second world war. And within this expansion, he further laments the relative and still now growing reach of what may be treated as matters of law over politics.

On Lord Sumption’s analysis, political questions are importantly bound up in processes and systems that are characterised by messiness, compromise and fudge, meaning they are not apt for resolution—practically or in principle—through application of law by judges. Similarly, he argues that matters of personal morality, understood within a liberal framing that—broadly speaking—provides and protects self-regarding decisions that do not harm others, are not appropriately addressed through the methods of public decision-making provided by courts of law.

In short, Lord Sumption may be represented as espousing a libertarian political morality that would assign (or recognise as fundamental) high degrees of freedom and responsibility to individuals, with a commensurately small reach for government. Insofar as the state and its institutions have their place, the courts’ role is one that reflects a narrow institutional competence, with matters that are better (within his framing) considered political being addressed elsewhere. Such a position indicates a powerful role for individual rights; side constraints against government interference with basic freedoms. However, even with their predominant protection of civil and political (‘negative’) rights, Lord Sumption opposes the evolving nature and form of human rights as found in and adjudicated under the European Convention on Human Rights (ECHR); in UK law through the Human Rights Act 1998.

Overall, Lord Sumption’s outlook on law, politics and morality therefore gives enduring importance to individual responsibility, recognises moral pluralism, and accordingly holds that law is wrongly used as a vehicle to achieve—or impose—‘social and moral conformity’ (p 8).^18^ Liberty, within his arguments, ought to be prized over security; and law ought not to enforce a single moral outlook in answer to questions where moral consensus is absent and the decision is self-regarding. In line with these views, it is perhaps unsurprising that he should have been a strident and prominent critic of restrictions that have been implemented in response to the coronavirus pandemic. However, as I will now go on to explore, such a position appears stridently at odds with moral and political considerations that he chose to draw into law itself through his decision in *Nicklinson*.

## Lord Sumption on the equal, inherent and unquestionable value of life: *Nicklinson*, the sanctity of life doctrine and the need to protect people who are deemed to be vulnerable

In 2014, the UK Supreme Court gave its judgment in the case of Tony Nicklinson. (It should perhaps be noted that before the appeal was heard, Mr Nicklinson had ended his life through refusal of food, given his despair at an earlier refusal by the courts to allow him to end his life as he would choose.^22^) Mr Nicklinson’s case challenged the lawfulness of the outright ban on assisting suicide in England and Wales, enshrined in section 2 of the Suicide Act 1961, on the basis that it breached Article 8 ECHR, which protects the right to respect for private and family life. In essence, the argument was that the blanket nature of the legal prohibition was incompatible with the rights of people who sought assisted dying because of profound suffering, who had an autonomous wish to die, but who required assistance to end their life. Interference with Article 8 rights may be justified if it is proportionate. However, it was argued that the legitimate aims of the Suicide Act (protecting people who are vulnerable) could be met while allowing exceptions for people in tragic situations such as Mr Nicklinson to access legally-regulated assisted dying. The case raised complex constitutional questions, as well, of course, as addressing one of the perennially controversial debates within bioethics and political morality.^23 24^ As such, in the Supreme Court nine—as opposed to the usual number of five—judges heard the case; among them Lord Sumption.

In total, the judgments of the Justices of the Supreme Court run to 366 paragraphs. The judges’ opinions and reasoning differ, with no single majority position against which to characterise the basis of the overall decision to dismiss Mr Nicklinson’s appeal. Nevertheless, Lord Sumption’s reasoning has had significant legal impact in subsequent litigation on assisted dying, including in relation to the matters that I explore in this paper (see for example paragraphs 91–94 and 98–113 of Noel Conway’s 2017 assisted-dying case).^25^ In what follows, I discuss two core parts of Lord Sumption’s judgment: his reasoning based on the inherent value of human life and how this should be respected at law; and his reasoning based on protecting people who are considered to be vulnerable.

### The value of life: inherent and beyond question

Lord Sumption introduces his judgment in *Nicklinson* in the following terms:

English judges tend to avoid addressing the moral foundations of law. It is not their function to lay down principles of morality, and the attempt leads to large generalisations which are commonly thought to be unhelpful. In some cases, however, it is unavoidable. This is one of them. (paragraph 207)^14^

Given Lord Sumption’s views on moral pluralism, individual responsibility, and the ill situation of judges to address questions of personal morality (as he conceives it), the framing in the initial two sentences is consistent with his general philosophy. But where he takes things with the final two sentences is accordingly striking and strange. Despite what Lord Sumption says here, on his own terms in finally addressing the question, laying down principles of morality would have been eminently avoidable. Consistent with his views, as presented above, the determinative reasoning of his judgment holds that Parliament, rather than the courts, is the properly placed institution to establish law and policy on the complex and morally-contested question of assisted dying. So given this, why he would decide to advance a process of legal reasoning by reference to morality is unclear. The core of his legal determination does not require it. As such, there is no explanation, for example, in suggesting that he was bound to seek to identify and address the moral foundations of law; to reason as he did given moral content found within positive law that Lord Sumption, as a judge, had to follow. Furthermore, the diverging judgments within *Nicklinson* evidence the lack of a clearly preclusive moral position within positive law here. Lord Sumption’s calling on himself to address questions of morality within his judgment, where morality does not even go to the core of his decision, accordingly seems to be an example precisely of the sort of judicial overreach that he castigates. His electing to approach the case on these terms thus invites particular interest in how he presents his ideas.

Lord Sumption’s judgment proceeds to explain the importance of moral values. He says: ‘There are some moral values, of which the state is the proper guardian, with no rational or utilitarian justification’ (paragraph 208).^14^ Their significance, he posits, emanates from their being ‘fundamental to our humanity and to our respect for our own kind’ (paragraph 208).^14^ Autonomy is one such value, he avers, before holding:

There is, however, another fundamental moral value, namely the sanctity of life. *A reverence for human life for its own sake is probably the most fundamental of all human social values*. It is common to all civilised societies, all developed legal systems and all internationally recognised statements of human rights. (paragraph 209)^14^ (emphasis added)

Lord Sumption goes on directly to cite approvingly *dicta* from Hoffmann LJ’s decision in the Bland case, stating ‘we have a strong feeling that there is an intrinsic value in human life, irrespective of whether it is valuable to the person concerned or indeed to anyone else’. (*Airedale NHS Trust v Bland*, p 826)^26^ Lord Sumption ends his quotation with Hoffmann LJ’s reflection on the ‘feelings’ that underpin this view of the value of life: ‘What matters is that, in one form or another, they form part of almost everyone’s intuitive values. *No law which ignores them can possibly hope to be acceptable*’ (p 826)^26^ (emphasis added).

Lord Sumption’s position here is clear. For reasons that I have suggested appear gratuitous, he chooses to advance a position rooted in morality rather than simply speak to institutional competence and constitutional principle. And in so doing, he proposes to identify (‘probably’) the *most fundamental* moral principle; the sanctity of life. He represents this principle not (I borrow here from John Keown’s terms) as a ‘vitalistic’ ethic that provides that life should be preserved in any circumstance no matter the cost.^27^ Rather, it is imposed as a principle that holds that all life is intrinsically valuable, that this value is undiminished by anyone’s effort at evaluation of it, and that to be morally legitimate it is a principle for which laws must—in substance, form, and application—show consistent respect.^27^ It is a general philosophical claim about the moral foundations of law, and is framed as a—as probably *the most*—basic moral proposition of ‘acceptable’ law in this jurisdiction. In the following section, I consider how this moral position relates to a second, distinct ethical aspect of Lord Sumption’s reasoning in the case.

### The value of life: protecting persons who cannot protect themselves

Despite Lord Sumption’s general disdain for state activity that prioritises security over liberty, he invites himself, in his decision in *Nicklinson*, to rest a substantial level of argumentative weight on ‘the problem of the effect on other vulnerable people’ if the law were changed to allow assisted dying (paragraph 215).^14^ This problem is the basis of the legitimate aim that is addressed through a general criminal ban on assisting suicide. The argument here is formally and practically distinct from considerations concerning the sanctity of life (as Lord Sumption clearly recognises), and is a familiar source of discussion within bioethical debate.^28^ The argument holds that even if we accept the idea that some instances of assisted dying are—as it were, on their own terms—acceptable (say in instances of autonomous choice combined with the decision being based on profound and unavoidable suffering), an outright ban is nevertheless needed because having an exception will lead to the wrongful deaths of persons who ought not to receive assisted dying (say because they have been pressured by cynical family members).^29^

Unlike the sanctity principle as presented above, this rationale does require a balancing exercise (so at least something approaching ‘a rational or utilitarian calculation’). That is true even if the calculus were that one wrongful death would be sufficient to justify prohibiting all instances of ‘legitimate’ assisted suicide. Of note here, even while arguing that this ought to be an analysis and decision for Parliament, Lord Sumption goes out of his way to explain clearly that he finds the position persuasive. He expresses in lengthy terms how and why the freedom of one person, however important that freedom may be, should not come at the cost of freedom apparently being exercised by others who may in fact be compelled to act against their own interests:

The vulnerability to pressure of the old or terminally ill is a more formidable problem. The problem is not that people may decide to kill themselves who are not fully competent mentally. I am prepared to accept that mental competence is capable of objective assessment by health professionals. *The real difficulty is that even the mentally competent may have reasons for deciding to kill themselves which reflect either overt pressure on them by others or their own assumptions about what others may think or expect*. The difficulty is particularly acute in the case of what the Commission on Assisted Dying called ‘*indirect social pressure*’. (paragraph 228)^14^ (emphases added)

It is perplexing that he should give these views such detailed and forthright prominence. Consistent with his own philosophy, it is unsurprising that Lord Sumption would not want to rule on the matter as a question of law, and would leave any change to the legal *status quo* to Parliament. Yet it is surprising that he should therefore use his judgment to advance a position that is ultimately one of policy and social ethics: namely, that protection of the life of people who are perceived to be vulnerable—and to emphasise again, he is referring to adults who do *not* lack capacity at law—is a justification for interference with liberty.

This, combined with his reflections on the sanctity principle, makes great swathes of Lord Sumption’s judgment in *Nicklinson* quite baffling: the pronouncements of a prolife, antichoice morality appear unwarranted given the key issue being one of institutional competence (and so the broader discussion a *political* overstep by a judge?). They also seem very much at odds with his own heavily liberty-prioritising position on social ethics. When explaining that both the moral contestability of assisted dying and the weighing of risks make it a matter for Parliament not the courts, he says of judges that the ‘imposition of their personal opinions on matters of this kind would lack all constitutional legitimacy’ (paragraph 230).^14^ Yet still, within the same section of his judgment, he goes on to say:

I have not dealt with the possibility that the present state of the law might also be justifiable under article 8.2 for the protection of morals. That is because the point was hardly argued, and because the protection of health seems to me to be a sufficient justification. But I would certainly not rule it out. The criminal law is not a purely utilitarian construct. Offences against the person engage moral considerations which may at least arguably be sufficient justification for a general statutory prohibition supported by criminal sanctions. (paragraph 235)^14^

The *Nicklinson* judgment is replete with profound claims of moral principle on the part of Lord Sumption. His final determination is that Parliament has decided how to regulate assisted suicide, and that it is the right institution to have done so. Yet, as the above passages show, he chooses nevertheless to advance two forthright moral propositions. First, that if the law is to be legitimate, inherent to it must be respect for the sanctity of each human life, regardless of what value anyone might place on it (with a particular need to guard against third parties’ assessments of the value of a person’s life). Second, that as a matter of policy one person’s liberty might be constrained for the protection of another (even where that other is an uncoerced adult with positively-confirmed decision-making capacity).

As already suggested, with these points explained it is interesting to ponder how his judgment sits relative to his general views on public ethics and of the role of judges. However, it is both interesting and pressing to contrast and then question the apparent distinctions that have emerged between Lord Sumption’s philosophical commitments in *Nicklinson* and his unending and deeply critical comments on the restrictions regulations that have been designed (however effectively in any given instance) to limit the spread of coronavirus in the UK.^13^ Notably, of course, such laws are presented as necessary by elected policy-makers who have heard multiple sources of evidence, weighed distinct considerations, and determined that they are required to protect life (including through preventing the NHS from being overwhelmed), and with a particular view to persons who may be vulnerable. As explained in the next section, Lord Sumption in this instance expresses vanishingly little concern for considerations of vulnerability to pressure, and disavows ideas about the sanctity of life.

## Liberty over security: Lord Sumption on the unequal, contingent and questionable value of life under coronavirus restrictions regulations

As discussed in the second section of this paper, Lord Sumption’s views on legitimate government are well framed as libertarian, small state and endorsing only a very limited role for law. In line with such views, since the emergence of coronavirus restrictions regulations in the UK he has been a strident critic of ‘lockdowns’ and associated measures. In a newspaper article published on 26 March, 2020, querying the legality of the implementation of the first nationwide lockdown, he wrote:

As the technical and administrative capacities of the state expand, so people demand more of it in their constant pursuit of security. The state must, if it can. Sometimes the state must, even if it cannot.^30^

Within that article and many others, and in other forms of media appearance, Lord Sumption has explained forcefully his views that the state cannot provide security against the coronavirus, that its efforts to do so are causing greater harms than would eventuate without restrictions regulations, and that anyway such regulations represent a manifestly unjustified legal interference with individual liberties. He has chided the government slogan ‘Protect the NHS’, saying: ‘The NHS is there to protect us, not the other way round.’^31^ He writes: ‘It is our business, not the state’s, to say what risks we take with our health.’^31^ (See also his argument referenced here.^32^)

As we have seen, in *Nicklinson* Lord Sumption expresses in lengthy detail his support for blanket restrictions on individual freedoms—even in instances of individuals’ decision-making capacity having been positively affirmed—in order to provide general protections against the fatal outcomes of overt pressure from others, questionable individual assumptions about other people’s expectations, or ‘indirect social pressure’. However, in the context of coronavirus, he seems profoundly and unquestioningly committed to the view that there are no moral difficulties to address concerning the meaningfully voluntary assumption or avoidance of risk by people who may be vulnerable. Lord Sumption has even framed his concerns regarding this point against a lament about a fear—as he puts it, ‘an irrational horror’^33^ —of death, and a failure of social acceptance of, and individual control over, death:

Today death is the great obscenity, inevitable but somehow unnatural. In the midst of life, our ancestors lived with death, an everpresent fact that they understood and accommodated. They experienced the death of friends and family, young and old, generally at home. Today it is hidden away in hospitals and care homes: out of sight and out of mind, unmentionable until it strikes.^33^

The bluntness and blanket nature of public health laws within the pandemic, he argues, ‘represent an interference with our lives and our personal autonomy that is intolerable’ and, however valuable the ends might be that they aim to achieve, their effect ‘is to treat human beings as objects, mere instruments of policy.’^33^

There are, I would suggest, overwhelming tensions between Lord Sumption’s moralistic approach to determining the bounds of people’s rights that he allowed to be drawn into law in the *Nicklinson* case, and his alternative moralistic approach as a commentator on COVID-19. There appears to be a radical variance in his view. As he has put it in a further anti-restrictions regulations piece: ‘Legal coercion is indiscriminate, whereas the virus discriminates.’^34^ The same might, of course, be said in relation to s.2 of the Suicide Act 1961 and being afflicted with conditions such as motor neurone disease. So it is startling, especially given what we see in his *Nicklinson* judgment, that Lord Sumption would say:

To justify their policies and command submission, ministers have to resort to fear, the classic tool of despotic regimes. They trumpet the small number of ‘long COVID-19’ cases. They wail like Old Testament prophets about the terrible plague that is on us. *They utter ugly threats about ‘killing Granny’.* 34 (emphasis added)

It is not clear how such strident critique would apply with less vigour to his judgment in *Nicklinson*, which has, as noted, gone on to shape the direction of subsequent end-of-life law. When it comes to actively choosing suicide within a regulated and policed assisted-dying system, Lord Sumption, cites the compelling need to protect people who may be vulnerable even while they still demonstrably enjoy decision-making capacity (‘an ugly threat about killing Granny’?). Yet against inadvertently contracting COVID-19, whether, for example, because of social pressure to attend particular places or events, or, for example, because of practical necessity given conditions of receiving care, there is cynicism rather than anything like equivalent consideration given to the issue that he offered in *Nicklinson*. Lord Sumption makes comments such as:

People are making their own judgements, guided by their own vulnerabilities and their own tolerance of risk. Left to themselves, people can manage this virus better than [Prime Minister] Boris Johnson and [Secretary of State for Health and Social Care Matthew] Hancock because they can fine-tune their precautions to their own situation and that of the people around them.^34^

There is no question within the position advanced that people may just actually not be able to avoid the risk. Nor does Lord Sumption seem to worry that people may, by virtue of their vulnerability, suffer ‘indirect social pressure’ to allow themselves to be exposed to the virus.

Insofar as his position rests on his claims about the practical futility of the regulations and the (anyway) unjustified interference with individual rights, there may be some (for want of a better term) higher-order consistency with Lord Sumption’s position in *Nicklinson*. Nevertheless, there remain some apparently irreconcilable inconsistencies in the framing, reasoning and application. Furthermore, Lord Sumption has chosen to promote views that are anathema to the sanctity position that he said he was compelled to present as fundamental in *Nicklinson*. Speaking on the BBC’s *The Big Questions* in January 2021,^35^ he articulated the view that lives were of relative value: indeed, he explained to a stage-4 cancer patient that her life was ‘less valuable’ by virtue of her situation, and that his children’s and grandchildren’s lives were ‘worth more [than his own] because they’ve got a lot more of it ahead’.

Lord Sumption subsequently sought to justify this position on the basis of a distinction in ‘context’, and by saying that he never promoted ‘the sanctity of life as an absolute value that prevails over absolutely everything else, always.’^36^ However, he seems here—one way or another—not to understand what he has argued. The claim about context does not work: the sanctity principle as a matter of law and morality is a universal and basic principle; as he said in *Nicklinson*, it is fundamental and general. The claim about absolutism misrepresents the principle: advocates of the sanctity principle (the coherence of whose position I note in passing I have been critical^37 38^) do not argue that it requires that life be preserved no matter what (‘vitalism’). Rather, just as Lord Sumption argues in *Nicklinson*, the sanctity principle permits for life-ending decisions, but at the same time it debars the state (or actors such as doctors) from placing differential values on life instead of recognising all lives as having universal, equal and inherent value.

Lord Sumption’s foundational position in the context of coronavirus regulations, as in his broader philosophical musings, seems to be one where (almost certainly) *the* foundational principle that makes *living* valuable is individual liberty (not the sanctity of life in and of itself). He accepts that side constraints must be placed on liberty, and in principle that this may be understood with reference to forms of harm (to others), nevertheless accommodating a toleration of risk and accepting a need to conduct a balancing exercise (‘a rational or utilitarian calculation’). In approaching that balancing in relation to coronavirus restrictions, however, he engages in a mode of practical reasoning that is antithetical in approach, form, substance, and even rhetoric to that which he has enshrined in law through his judgment in *Nicklinson*. The challenge of his inconsistency speaks both to the quality that we then ought to perceive in his moral and policy pronouncements as a judge in that case, and to the quality of his contributions to public debates on how best to respond to the coronavirus pandemic.

## Conclusions

Although it was unnecessary given the basis on which he ultimately reached his decision in *Nicklinson*, Lord Sumption in that case expressed a reverence for the moral principle of the sanctity of life as a—perhaps the most—fundamental social value, and one that must be upheld through law. Equally unnecessarily, he also provided an apparently subtle analysis of how harmful vulnerabilities may manifest themselves, on the back of which he held that public safety (framed in terms of public health) should take precedence over individual liberty. Those points of reasoning have proven to be important aspects of subsequent litigation concerning assisted-dying laws.^25 39^

Despite his election to entrench these (as he would frame them) moral and political positions within his own legal decision, making them ripe for incorporation into subsequent legal judgments, they clearly appear to grate against his general—prominent—commentaries on law, politics and social ethics. And the way in which the dissonance presents itself is all the more marked in the context of his critiques of coronavirus restrictions regulations. Here, Lord Sumption’s intolerant and summary dismissals of policy aims and rationales suggest that his own reasoning in *Nicklinson* might be well characterised as gratuitous moralising, and even fear-mongering rhetoric dressed up as—again gratuitous—policy analysis and evaluation.

Not only does Lord Sumption’s approach to balancing security against liberty when writing as a commentator call into question his impacts on law when acting as a judge, he has also managed in his deliberations on coronavirus to challenge his own appraisal of the inherent value of life. It may, of course, be the case that his ideas have changed since *Nicklinson*. But if so, it is problematic from the very perspective of his own critical works on judging and judicial overreach that he took the opportunity unnecessarily to expound so heavily on the back of moral and political reasoning where these need not have arisen as questions of law. Somewhat paradoxically, the closest that I see to squaring the circle may be found in whatever underpins his arguments that people might, for moral reasons, ignore—break—the law: both in relation to restrictions regulations and assisted-dying laws.^40^ (He also indicates in his judgment in *Nicklinson* that in different times unlawful but unchecked physician-assisted suicide would have been (acceptably?) viable^i^). That a former Justice of the Supreme Court would express views in this direction raises various questions. But in any event, his doing so does not obviously allow us to overcome challenges in surpassing the apparent points of incoherence in Lord Sumption’s positions on the values of life, liberty and security, and their relationship with law.

## Footnotes

Lord Sumption says in *Nicklinson*, at paragraph 210: ‘[I]t is more difficult in modern conditions for intensely personal end-of-life choices to be made informally, within the family and with the support of a trusted medical practitioner. The medical profession, for wholly understandable reasons, is less willing in a transparent, highly regulated and litigious world to take the responsibility for cutting life short or helping someone else to do so, without an assurance of immunity which in the present state of the law is impossible to give.’
